# Maternity and Mothering

**Published:** 1911-12-02

**Authors:** 


### MATERNITY AND MOTHERING.

(Inquiries and Correspondence are invited.)

### Rest and Exercise after Confinement.

TAKING the various stages of the puerperium, or lying-in period, in their due chronological order, there are first to be considered the arrangements of the first day or two following delivery, which may fitly be regarded as a time of recovery from the exhaustion, stress, and anxiety of the severe ordeal from which the patient has successfully emerged. Complete recuperation and recovery require, of course, much longer than this; but for convenience the period up to the establishment of lactation may well be considered as especially one of restoration and reinvigoration, by sleep and rest, in normal cases, which are alone under discussion now.

As was shown in the previous article, this period is spent supine by most patients; and some doctors even object to the use of a pillow for the head during the first twelve hours. The latter restriction is, in the writer's judgment, a stupid and unnecessary hardship to the patient; and as a matter of fact it is steadily declining in popularity with the profession. Some physicians allow the lateral posture to be assumed, should the patient prefer it; and really it is difficult to see any valid objection to this, the natural position for rest and slumber.

Patients who are confined entirely to the supine posture often find considerable hindrance during the first twenty-four hours to the function of micturition. Lying flat on the back, the force of gravity has to be overcome before the stream can be expelled; and the recently relieved over-distension of the rectus and other abdominal muscles often renders them useless for some little time as far as assisting in the performance of micturition is concerned. To get over this trouble, it often suffices to roll the patient over on to her face, or even to allow her to assume the hands-and-knees posture for a minute; for then gravity is a help instead of a hindrance. Such permission is not granted in this country for at least ten hours after delivery, even by those who favour the utmost liberty in this direction. Some authorities object to these instructions, and order the application of hot compresses to the lower part of the abdomen; some even prefer the use of the catheter to any disturbance of the patient. The passage of a catheter, however, should properly be regarded as a surgical operation; for it has serious drawbacks and complications of its own.

The reason why greater freedom of movement is not encouraged is the fear of that most terrible of calamities, the occurrence of embolism. Few tragedies are more sudden, more pitiful, than the rapid transition from apparent health to instant death occasioned by this accident; and the barest possibility of it should be enough to call forth every precaution that will avail against it. In Java, it is reported, the native custom is opposed to rest in bed after delivery, and embolism, thrombosis, and prolapse are comparatively frequent results.

Notwithstanding this object-lesson, some Continental obstetricians actually allow the erect posture on the first day after delivery. Thus Von Alvensleben in 1908 reported his experience with a hundred patients who were allowed up from the first to the fourth days. All those who had never previously been confined were allowed to get up for an hour on the first day; and as no ill-effects were noted, but on the contrary, a more rapid recovery of appetite, etc., he afterwards gave all patients whose confinements had been normal, the same latitude. Even severe hæmorrhage after the labour was not a contraindication of his daring innovation, though deep lacerations of the parturient canal were so considered. On the first day the patients took a few steps to a chair, in which they sat up for an hour. The next day they were required to walk once up and down the room; on the third day this was repeated twice; and on the fifth day, six hours would be spent out of bed. Six patients out of the hundred had to be taken back to bed for faintness and similar symptoms; ninety are said to have exhibited no ill-effects at all, but on the contrary benefits.

This system is here quoted not as an example to be copied but merely to show to what lengths some men have gone without being overtaken by disaster. Without imitating these drastic, and (as Englishmen think) dangerous experiments, it at least behoves us to keep an open mind and to admit that a little gradually undertaken and carefully supervised muscular movement may perhaps profitably be encouraged before the tenth day. Thus it is comfortable to the patient, and seems to be perfectly harmless, to allow her (everything progressing normally) to be propped up about the fourth or fifth day; at first a little, and then more until the sitting posture is reached about the seventh or eighth day. Such a routine does not dispose to retrodeviation of the uterus, or to subinvolution. It must not, naturally, be regarded as a cast-iron policy to which all patients are to be forced, willy nilly, to adhere. It must be interpreted with discretion, and allowance must be made for the factors of each individual case. If the red lochia persists in unusual quantity, and there is no cause such as retained secundines to account for this, it is advisable to insist on complete rest in bed until, at least, the uterus is so far involuted as to be no longer palpable per abdomen when the bladder is empty.

The further question arises as to the desirability or otherwise of exercises to be carried out by the patient during her stay in bed. According to the same German authors who advocate the very early rising principles to which we have referred, such exercises are extremely beneficial in promoting appetite, circulation, and hence a speedy convalescence. Without denying the possibility of this, it is doubtful whether in this country such treatment is feasible; the British clinician has hitherto paid scant attention to these well-meant suggestions; and probably he is wise in his generation in being thus slow to introduce innovations which his patients might very easily resent.

### The Endowment of Motherhood.

When Mr. H. G. Wells's "New Machiavelli" appeared his critics discussed at length the moral problem there presented—whether a man should be excluded from public life because of an open lapse from domestic virtue. But they do not seem to have noticed a fine touch of technical skill in the fact that Remington's career is cut short just when he has won great popularity by advocating the endowment of motherhood. For this break saves the author from the necessity of defining what the endowment of motherhood is, and leaves only a mass of rhetorical statement with which most people will agree. But Mr. Wells is a social philosopher as well as an artist, and one would fain know just what he means by the endowment of motherhood, and how he would suggest its being accomplished.

It may be said that the maternity benefits promised under the Insurance Bill are a commencement of such legislation, but it is a very small beginning. One can hardly call thirty shillings, all of which will be spent within the first month of the child's life, an endowment. The bribe is hardly large enough to make provident people indulge in a larger family. The fundamental intention of endowing motherhood is rather the improvement of the race than the temporary comfort of the mother; and thirty shillings will not go far in ensuring that. Ideally, of course, no marriage should take place where the contracting parties cannot show a sufficient capital of health, industry, and prospects of success to justify their becoming parents; but as a matter of practical fact there is little hope of such an ideal being realised, nor, even if it were here, could we be sure that those to whom the marriage licence was refused would live in that chastity which would be necessary if the purpose of the refusal was not to be evaded.

Taking marriage as we find it to-day, then, what can we do for the endowment of motherhood? It is difficult to say, because the classes in which motherhood is going out of fashion are those which do not really stand in urgent need of endowment. A great deal of vituperation is launched against the childless women among the richer classes, who are supposed to prefer pet dogs to babies, and pleasure and irresponsibility to both. But even if all that is said were true, the rich form but a small proportion of the population, and their comparative childlessness is not a great matter. The people who say that they "can't afford children" are neither the rich nor the poor, but the poorer members of the middle class, the clerks and the professional people with incomes just over the income-tax limit. These people are not eligible for Mr. Lloyd George's thirty shillings and would find small consolation in it if they were. How are the mothers of this class to be endowed? They do not want money for their children's food, but they want perhaps more than anything such large educational advantages as Germany gives all her people for little or nothing.

As for the lower classes, are they to be helped by money doles? That is in many minds the idea of the endowment of motherhood. It is the obvious, the simple way of achieving the desired end, and if it were proposed to make a grant in aid of the maintenance of every child in the kingdom under the age of sixteen, those who opposed it would probably be held to be protesting merely out of regard for their own pockets. And many people would welcome the scheme as a brilliant proof of the humanitarianism of the twentieth century. But economic heresies are like religious ones: they appear and reappear in succeeding generations. The method of giving doles to the poor in proportion to their families was tried under the old Poor Law, and the abuses to which the system led helped to make the Poor Law which succeeded it all the more harsh. The expectant parents warned the overseer of the poor of the near advent of a new little pauper, as a matter of course. And the result was that as doles grew wages declined. Men's wages were fixed by the law by which women's are fixed still—that is, on the basis of the single man, or at most the childless couple—the provision for babies was expected to come out of the public purse. A peculiar evil developed in the case of illegitimate children. Magistrates showed themselves exceedingly ready to take a woman's word as to the parentage of her child—especially if she put the blame on a man who could afford to pay for its maintenance, as this saved the rates, and children thus became an investment—with the strange result that often a woman with several illegitimate children was looked upon as rather an eligible bride than the reverse.

A better endowment of motherhood would be to make it compulsory for a man to give a rightful proportion of his earnings for the maintenance of his wife and children. This proportion might perhaps be regulated by the size of the family; the master of the house would generally see to it that his share did not fall too low, and a slack acceptance of low wages would be the last thing thought of. For the rest, greater care in house-planning, the abolition of slums, and severer laws against adulteration of food, severely administered, would do much to keep alive the children that are already here. A population must be judged by its efficiency as much as by its size, and the cradle whose occupant dies before the end of his first year is no great boon to a nation, however often it may be re-filled. It is better for a woman to bring up three children to strong, sound manhood and womanhood than to bear ten who die before reaching adult life.

It is not true that the modern woman seeks to avoid child-bearing. The women of to-day take a more serious view of life than their mothers did, and honour motherhood no less that they enter upon its responsibilities less recklessly than their predecessors. They do not need to be bribed with an endowment of motherhood, but they need to be encouraged by an improvement in the conditions of motherhood. The Midwives Act was a true endowment; our district nursing organisations are an endowment; an abundance of good water and fresh air are endowments; the assurance that the milk a child drinks and the bread he eats are pure and sound is an endowment: and it is such endowments that help without demoralising the recipient.

